# Anatomical assessment of local recurrence site in breast cancer patients after breast reconstruction and post-mastectomy radiotherapy: implications for radiation volumes and techniques

**DOI:** 10.1007/s11547-024-01812-z

**Published:** 2024-04-11

**Authors:** Viola Salvestrini, Marianna Valzano, Icro Meattini, Carlotta Becherini, Luca Visani, Giulio Francolini, Ilaria Morelli, Niccolò Bertini, Lorenzo Orzalesi, Marco Bernini, Simonetta Bianchi, Gabriele Simontacchi, Lorenzo Livi, Isacco Desideri

**Affiliations:** 1 Radiation Oncology Unit, Oncology Department, Careggi University Hospital, Viale Morgagni 85, 50134 Florence, Italy; 2 Department of Experimental and Clinical Biomedical Sciences “M. Serio”, University of Florence, Florence, Italy; 3 Breast Surgery Unit, Careggi University Hospital, Florence, Italy; 4 Division of Pathological Anatomy, Department of Health Sciences, University of Florence, Florence, Italy

**Keywords:** Post-mastectomy radiotherapy, Target volume, Local regional relapse

## Abstract

**Introduction:**

Post-mastectomy radiotherapy (PMRT) improves local control rates and survival in patients with adverse prognostic features. The dose coverage to target volumes is critical to yield maximum benefit to treated patients, increasing local control and reducing risk of toxicity. This study aims to assess patterns of breast cancer relapse in patients treated with mastectomy, breast reconstruction and PMRT.

**Methods:**

Breast cancer patients treated with PMRT between 1992 and 2017 were retrospectively reviewed. Clinical and pathological characteristics of patients were collected. Recurrences were defined as “in field,” “marginal” or “out of field.” Survival analyses were performed in relation to progression-free survival (PFS) and overall survival (OS). Correlation between baseline features was explored.

**Results:**

Data of 140 patients are collected. After a median follow-up time of 72 months, median PFS and OS of 63 and 74 months were detected, respectively. Neoadjuvant chemotherapy, lympho-vascular space invasion (LVI) and size of primary tumor were all significantly associated with worst PFS and OS. Ten patients developed local recurrence: 30% "in field," 30% marginal recurrences, 20% "out of field" and 20% both “in field” and “out of field.” No recurrence was detected under the expander, 80% above the device and 20% patients relapsed on IMN chain. The mean distant relapse-free survival was 39 months. Overall, 39 of 140 patients developed distant metastases.

**Conclusions:**

The onset of local–regional relapses occurred mainly above the expander/prosthesis, underlying the importance of inclusion of the subcutaneous tissues within the target volume. In order to refine new contouring recommendations for PMRT and breast reconstruction, future prospective studies are needed.

## Introduction

Higher T and N stage, younger age at diagnosis, estrogen receptor (ER)-negative disease and presence of extracapsular extension are crucial factors associated with post-mastectomy risk of locoregional recurrence [1, 2]. Post-mastectomy radiotherapy (PMRT) showed to improve local control rates and survival in patients with adverse prognostic features (e.g., T4 tumors, positive margins, > 3 positive nodes, estrogen receptor-negative disease or young age) [2]. According to the literature, standard indications for PMRT are stage III disease, T3N0 (if high-risk features) and node-positive disease (debated the number of positive nodes) with remarkable benefit in patients with > 3 axillary involved lymph nodes [3]. However, the optimal definition of target volumes and dose coverage are still debated in order to increase locoregional control and reduce unnecessary risk of toxicity. Indeed, the excessive inclusion of normal tissue in the field of treatment may increase risk of acute and late toxicity. In this regard, recent development of common contouring guidelines [4–6] helped to uniform target volumes definition and to reduce impact of interobserver variability, a potential confounding factor influencing dose coverage, dose to organs at risk and patient outcome after PMRT [8], especially in terms of quality assessment of clinical trials [9, 10]. Moreover, suboptimal coverage of the microscopic disease sites may lead to higher risk of recurrence. Advances in breast cancer radiation therapy (RT) have led to the introduction of 4D planning, intensity-modulated RT (IMRT) and volumetric arc therapy increasing dose homogeneity and normal tissue sparing [11].

The aforementioned critical considerations are of utmost importance especially if modern oncoplastic techniques (e.g., prepectoral or subpectoral tissue expander placement) prompt clinicians to tailor treatment based on clinical judgment. In this situation, the appropriate spatial identification of relapse patterns after PMRT has to be implemented in clinical practice. Therefore, this study aims to assess patterns of breast cancer relapse in a monocentric large series of patients treated with mastectomy, breast reconstruction and PMRT, in order to identify which target volumes are at higher or lower risk of recurrence after treatment, allow effective target coverage and organs at risk sparing.

## Materials and methods

Between 1992 and 2017, clinical records of patients with locally advanced breast cancer treated at the Radiation Oncology Unit, Oncology Department, of the Azienda Ospedaliero-Universitaria Careggi, University of Florence, were retrospectively reviewed. We included (1) stage IIA–IIIC breast cancer patients (2) aged > 18 years old (3) treated with mastectomy, axillary lymphadenectomy, post-surgical breast reconstruction with prepectoral or subpectoral tissue expander placement and PMRT. Regional nodal irradiation (RNI) was performed at clinician discretion according to baseline risk features. Data about age, disease stage at diagnosis (according to AJCC classification VIII edition), grading, estrogen and progesterone receptor (PgR) status, Ki-67 proliferative index, human epidermal growth factor receptor 2 (HER2) status, multifocality, multicentricity, presence of lympho-vascular space invasion (LVI) and treatments administered were collected and reported. Data about relapse detection site were retrieved from clinical reports, recurrences were defined as “in field,” “marginal” or “out of field” according to their position relative to chest wall treatment field and nodal target volumes, defined according to European SocieTy for Radiotherapy and Oncology (ESTRO) guidelines [7, 12]. All recurrences clinically detected within 1 cm from chest wall or within regional lymph nodes (when RNI was performed) were defined as “in field.” Recurrences detected between 1 and 2 cm from chest wall were defined as “marginal.” Finally, any relapse that occurred outside 2 cm from the treatment field within regional lymph nodes (when RNI was not performed) or outside from regional lymph nodes was defined as “out of field.” Data about site of recurrences in relation to tissue expander were collected. Survival analyses were performed in relation to progression-free survival (PFS) and overall survival (OS). The last follow-up and the cause of death were collected. Correlation between baseline features (use of neoadjuvant chemotherapy, grading, size of the primary tumor and LVI) was explored. Univariate analysis was conducted through log-rank test. A multivariable Cox proportional regression model was used to identify independent factors of specific events. All statistical analyses were performed using Medcalc® software.

### Surgical treatment

All patients underwent mastectomy and axillary lymphadenectomy, defined as the dissection of at least ten level I and II axillary nodes, according to the European Organisation for Research and Treatment of Cancer (EORTC) breast cancer group manual [13]. Post-mastectomy breast reconstruction with prepectoral or subpectoral expander placement was performed.

### Radiation therapy

Concerning PMRT, all included patients received a total dose of 50 Gy in 25 fractions. Patients underwent CT simulation with a breast board and supine position. Patients were scanned with 5-mm slices from mid-neck to mid-abdomen. The chest wall clinical target volume (CTV) was delineated on transverse CT images, and the planning target volume (PTV) was obtained by adding a 5-mm margin to the CTV. A two opposed tangential fields technique was used. A boost to surgical scar was administered in selected cases (e.g., tumor infiltrating the pectoral fascia). RNI was performed when at least four positive nodes were found at the pathological examination. Axillary nodal levels III–IV were treated with a nondivergent anterior photon field including the head of the clavicle medially and the coracoid process laterally. Inferiorly, the border was matched to the superior limit of the chest wall. Two techniques were used to cover internal mammary nodes (IMN), either a modified wide-tangent technique with the upper fields widened to include the internal mammary nodes, or a separate photon field angled to match the tangent fields used to cover chest wall CTV [14, 15].

### Systemic treatment

Adjuvant or neoadjuvant systemic therapies were administered in all patients. Chemotherapy included both anthracyclines and taxane regimens, trastuzumab for HER2-positive patients was used since 2005. Postoperative hormonal treatment was administered in ER-positive patients.

## Results

**Fig. 1:**
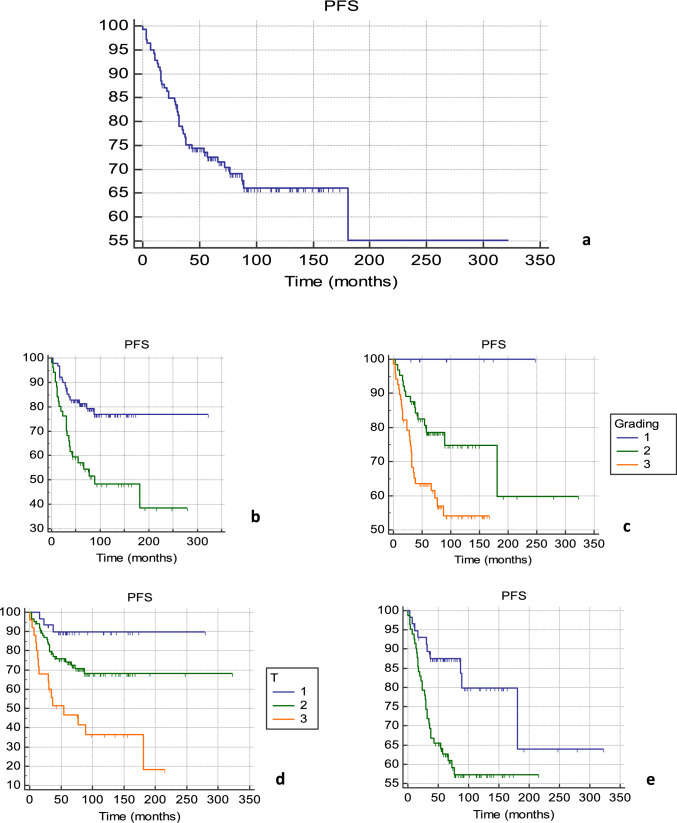
Progression-free survival (PFS). Mean PFS: 75.7 months (range 66,2–85,3; 95% CI). Median PFS: 63 months (range 54–75, 1; 95% CI) (**a**). PFS in patients not undergoing neoadjuvant chemotherapy (blue) and performing preoperative treatments (green) (**b**). Impact of grading on PFS (**c**). Impact of primary tumor size (T) on PFS (**d**). PFS in patients with (green) or without (blue) lympho-vascular space invasion (LVI) (**e**)

**Fig. 2:**
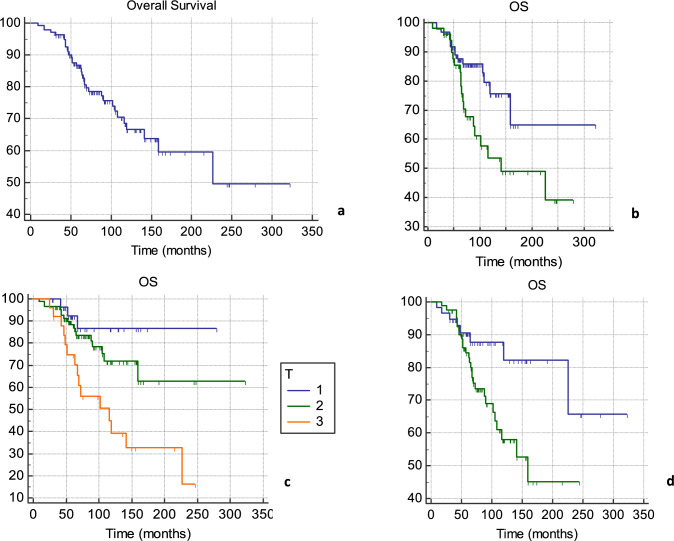
Overall survival (OS). Mean OS: 90.2 months (range 9–322; 95% CI). Median OS: 74 months (range 65–84.07; 95%CI) (**a**). OS in patients not undergoing neoadjuvant chemotherapy (blue) and patients undergoing preoperative treatments (green) (**b**). Impact of primary tumor size (T) on PFS (**c**). OS with (green) or without (blue) lympho-vascular space invasion (LVI) (**d**)

The individual characteristics of 140 patients are summarized in Table 1. Average age was 48.7 years (range, 30–71 years). The treatment characteristics of all 140 patients are listed in Table 2. Fifty-three out of 115 patients (46.1%) received letrozole, 51 patients (36.4%) received neoadjuvant chemotherapy, 105 (75%) adjuvant chemotherapy and, in 18 patients (12.9%), neo- and adjuvant treatments were performed. The whole cohort of patients enrolled in the analysis received radical mastectomy. Expander-based breast reconstruction was performed in 128 patients (91.4%), and 12 patients (8.6%) received definitive prosthesis. Notably, 105 patients underwent prepectoral breast reconstruction, and 35 patients underwent a subpectoral tissue expander placement. A Chi-square test (*X*^2^) of independence was performed to examine the relation between breast reconstruction type and the incidence of relapse. The relation between these variables was not statistically significant, *X*^2^ is 0.1436 and p-value is 0.7 (Table 3). One hundred and four patients received PMRT with RNI. After a median follow-up time of 72 months, median PFS and OS of 63 (range 54–75, 1; 95% CI) and 74 months (range 65–84.07; 95%CI) were detected, respectively (Figs. 1a and 2a). At univariate analysis, neoadjuvant chemotherapy, higher tumor grade, larger size of primary tumor and LVI were all significantly associated with worst PFS. Mean PFS was 78.01 months without neoadjuvant chemotherapy and 71.8 in the primary systemic therapy population (*p* = 0.0006); 110.48 months for G1 tumors, 81.5 for G2 and 66.9 for G3 tumors (*p* = 0.0078); 86.6 months for T1, 75.87 for T2 and 65.12 for T3 (*p* = 0.0001) and 87.3 months without LVI and 68.35 with LVI (*p* = 0.0039) (Fig. 1b–e). At the multivariate analysis, all the aforementioned factors showed to be independently associated with PFS (*p* = 0.005, *p* = 0.01, *p* = 0.02 and *p* = 0.02 for use neoadjuvant chemotherapy, tumor grading, LVI and tumor size, respectively). At univariate analysis, use of neoadjuvant chemotherapy, LVI and size of primary tumor were all significantly associated with worst OS. Mean OS was 85.7 months without neoadjuvant chemotherapy versus 74.95 in the population performing primary systemic therapy (*p* = 0.0282); 94.3 months without LVI versus 87.3 with LVI (*p* = 0.0023) and 90.4 months for T1, 88.12 for T2 and 81.26 for T3 (*p* 0.0011) (Fig. 2b–d). At the multivariate analysis, none of the factors was independently associated with OS. Extent of extracapsular extension (ECE), ER status and HER-2 status are not statistically associated with PFS or OS at univariate nor multivariate analysis.

**Table 1:** Individual characteristics of patients

Feature	Number	%
Age, years		
Age mean	48,7	
Range	30–71	
≤ 40	25	17,9
> 40	115	82,1
Primary tumor (*T*)		
* T*1	29	20,7
* T*1a	1	0,7
* T*1b	3	2,1
* T*1c	25	17,9
* T*2	85	60,7
* T*3	26	18,6
Lymph node status (*N*)		
* N*0	6	4,3
* N*1	31	22,1
* N*2	61	43,6
* N*3	42	30
Stage (AJCC edition VIII)		
IIA	5	3,6
IIB	16	11,4
IIIA	77	55
IIIC	42	30
Histologic tumor grading (*G*)		
* G*1	8	5,7
* G*2	64	45,7
* G*3	68	48,6
ER status		
Positive	114	81,4
Negative	25	17,9
Not available	1	0,7
PgR status		
Positive	101	72,1
Negative	38	27,2
Not available	1	0,71
Ki67 proliferative index		
≤ 20	53	37,9
> 20	78	55,7
Not available	9	6,4
HER2 status		
Negative	90	64,3
Positive	33	23,6
Not available	17	12,1
Multifocality		
Absence	51	36,4
Presence	89	63,6
Multicentricity presence		
Absence	47	33,6
Presence	93	66,4
Lympho-vascular invasion (LVI)		
Absence	58	41,4
Presence	82	58,6

**Table 2:** Treatment characteristics

Feature	Number	%
*Endocrine therapy*		
No	25	17,9
Yes	115	82,1
*Endocrine therapy regimen*		
Tamoxifen	23	20
Letrozole	53	46,1
Exemestane	7	6,1
Anastrozole	6	5,2
Exemestane + LHRH analog	3	2,6
Tamoxifen + LHRH analog	23	20
*Preoperative chemotherapy*		
Yes	51	36,4
No	89	63,6
*Surgery*		
Mastectomy	140	100
Axillary lymph node dissection	140	100
Tissue expander breast reconstruction	128	91,4
Definitive prosthesis breast reconstruction	12	8,6
*Postoperative chemotherapy*		
Yes	105	75
No	35	25

**Table 3:** Relation between breast reconstruction type and relapse

	Prepectoral breast reconstruction	Subpectoral breast reconstruction	
Relapse	7	3	10
No relapse	98	32	130
	105	35	140
Chi-square (*X*^2^) test = 0.1436 *P* = 0.72

### Patterns of local–regional recurrences

Overall, 10 patients (7.1%) developed local recurrence; average time to recurrence was 27 months (range 3–87). Average size of recurrence was 18.5 mm (range 6–30). Tables 4 and 5 present characteristics of patients with local–regional recurrences. Notably, the majority of the population with recurrence disease had T2 tumor (80%), N2 stage (60%) and stage IIIA (70%) at diagnosis. Most patients had aggressive biological features at diagnosis, like a Ki67 index ≥ 20% (60%) or a G3 tumor grading (80%). Recurrences were defined as "in field," "out of field" and marginal recurrences in 3 (30%), 2 (20%) and 3 patients (30%), respectively. Two patients had both "in field" and "out of field" recurrences. Concerning the site of relapse in relation to tissue expander, no recurrence was detected under the device, 8 patients (80%) had relapse above the device, 2 (20%) patients relapsed on IMN chain. After recurrence, four patients received local re-irradiation for an average total dose of 36.25 Gy (range 30–50 Gy), three patients received endocrine therapy alone, two patients underwent surgery and one patient was treated with chemotherapy. Three relapsed patients were still alive with no evidence of disease, one is alive with distant metastases, four died for metastatic disease and two died for other causes. The mean distant relapse-free survival (DRFS) was 39 months (range, 3–215 months). Overall, 39 of 140 patients (27.9%) developed distant metastases, and data are summarized in supplementary material (Table 5). Most patients with metastatic disease (51.3%) were treated with chemotherapy. At the time of the analysis, ten patients (25.6%) were still alive with metastatic disease, twenty-eight patients (71.8%) died for disease progression and one patient (2.6%) died for other causes.

**Table 4:** Local–regional recurrences

Feature	Number	%
*Recurrence*		
No	130	92,9
Yes	10	7,1
*Size*		
Mean, mm	18,5	
Range	Jun-30	
*Type*		
“In field”	3	30
“Out of field”	2	20
“In field” plus “out of field”	2	20
Marginal	3	30
*Site*		
Above expander	6	60
Above expander plus lymph node region	2	20
Internal mammary lymph nodes	2	20
Behind expander	0	0
*Local–regional recurrence treatments*		
Surgery	1	10
Chemotherapy (CHT)	1	10
Radiotherapy (RT)	1	10
Endocrine therapy (ET)	3	30
RT plus CHT	2	20
Surgery plus ET	1	10
Surgery plus RT plus ET	1	10

**Table 5:** Individual characteristics at diagnosis of patients reporting local–regional relapse

	Age	*T*	*N*	Stage	Grading	ER status	PgR status	HER2 status	Ki67 index	LVI
1	44	*T*2	*N*2	IIIA	G3	−	−	+	60	+
2	59	*T*3	*N*3	IIIC	G3	+	+	+	40	+
3	44	*T*2	*N*2	IIIA	G3	+	+	−	40	+
4	33	*T*2	*N*2	IIIA	G3	+	+	−	50	+
5	32	*T*2	*N*3	IIIC	G3	−	−	+	70	−
6	43	*T*2	*N*3	IIIC	G3	+	+	−	Not available	+
7	36	*T*2	*N*2	IIIA	G2	+	+	−	15	+
8	37	*T*2	*N*2	IIIA	G2	+	−	+	5	+
9	45	*T*3	*N*1	IIIA	G3	+	+	+	Not available	+
10	46	*T*2	*N*2	IIIA	G3	−	−	−	80	+

## Discussion

Over the past decades, the introduction of modern oncoplastic techniques has revolutionized breast surgical approaches. Modern advances in breast reconstruction have led to the transition from the traditional submuscular procedure to the new prepectoral implant-based strategy. Therefore, many experience have aimed to define the optimal breast reconstruction type for patients treated with mastectomy [16]. Recently, this new technique is increasing in the high-volume centers owing to its more minimal approach. Indeed, a retrospective analysis of 146 patients showed that immediate prepectoral breast reconstruction was effective and safe with five locoregional recurrences after 4 years of follow-up and a low acute and early-late complication rates [17]. According to our data, 25% and 75% of patients received subpectoral and prepectoral breast reconstruction, respectively. We did not find a statistically significant correlation between prepectoral procedures and the incidence of relapse. In line with the previous literature, our findings may support the adoption of the subcutaneous implant-based reconstruction as an effective procedure in terms of locoregional control. However, further studies comparing the prepectoral approach with the previous standard of care are strongly needed in order to evaluate long-term oncological and surgical outcomes.

**Fig. 3:**
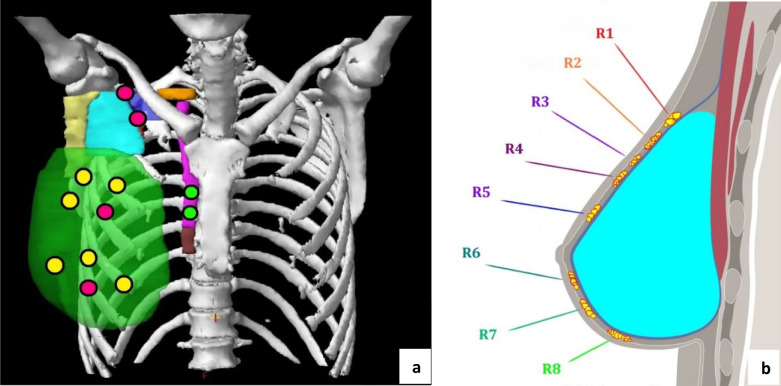
Site of chest wall recurrences: Relapse occurs above tissue expander (60%), above tissue expander and at the level of axillary nodes region (magenta) (20%) and internal mammary chain (green) (20%); modified from Gee et al. [20] (**a**). Spatial distribution of chest wall recurrences (**b**)

As expected, our results also confirmed the low rate of local–regional recurrence after PMRT and reported both in field and marginal/out of field recurrence. In this regard, many authors have focused their efforts on the investigation about correct target definition and dose coverage in this setting of patients [18]. To the best of our knowledge, a consensus recommendation for the optimal coverage of treated areas in breast contouring is still missing. ESTRO guidelines aim to minimize target volumes while RADCOMP recommendations, regarding patients potentially at greater risk of recurrence, delineated an enlargement of the treated area [7, 19]. However, relapses were significantly associated with biological aggressiveness of disease, suggesting that suboptimal target coverage may not be the only factor influencing risk recurrence. Of note, local recurrences in the present series were exclusively located above the expander, suggesting that target coverage of the subcutaneous tissue within skin and implanted device is critical for treatment outcome (Fig. 3 [20]). Moreover, under-prosthetic relapse is an extremely rare event, and dose coverage of implants, ribs and intercostal muscles should not be considered a critical issue from this point of view. Skin bolus remains an option in selected cases with a high risk of recurrence, to minimize build-up influence on target coverage. Randomized trials and meta-analyses confirmed the benefit of PMRT in terms of local–regional control (reducing the recurrence rate) and overall survival (OS) in patients with high-risk breast cancer [21–24]. Although the aforementioned results led to a growth in PMRT indications [23], this treatment is related to an increase in the complication rate (lymphedema, brachial plexopathy, radiation pneumonia, rib fractures, cardiac toxicity and radiation-induced malignancies) [25–39] and a greater risk of breast reconstruction failure [40–43]. For this reason, minimizing the exposure of organs at risk and selecting the target volumes at risk for local recurrence are a critical issue in this setting.

The limitations of the present experience are the retrospective nature of the analysis and the limited sample size.

Notwithstanding the limits of the present study, our data are in line with the current indication from ESTRO/ACROP guidelines recommending that after tissue expander reconstruction, only tissue in above the device should be included in the target volume, to reduce treatment complications. Only patients with under-pectoral placement of device with particular risk factors (e.g., large primary tumor, pectoral muscle or chest wall infiltration or poor response to neoadjuvant chemotherapy) may benefit from irradiation of under-prosthetic tissues [44].

## Conclusion

In conclusion, our real-life retrospective experience described the recurrence at the level of the chest wall as an extremely rare event in the case of PMRT. Both in field and marginal/out of field recurrences were detected, suggesting that some patients may recur due to the biological aggressiveness of disease rather than suboptimal coverage of target volumes. The onset of local–regional relapses occurred mainly above the expander/prosthesis, underlying the importance of inclusion of the subcutaneous tissues within the target volume. In order to refine new contouring recommendations for PMRT and breast reconstruction, future prospective studies are needed. However, the design of the ideal study to assess the relationship between treatment volumes and patterns of recurrences remains a challenge.

## Data Availability

Not applicable.
